# Iod. Potassium in Hydrocephalus

**Published:** 1850-09

**Authors:** Valentine Mott Satterlee

**Affiliations:** Green Bay, Wis.


### ARTICLE VIII.

*Iod. Pot. in Hydrocephalus.*—By Valentine Mott Satterlee, M. D., of Green Bay, Wis.

The following being quite an interesting case, and serving to show the benefit derived from the use of Iodide of Potassium, in cases of Hydrocephalus, which I have on several occasions treated successfully, I deem it my duty to report it that my brother practitioners may take advantage of the practice when similarly situated. Two weeks after the first attack, which was ushered in by convulsions, her condition was as follows: Pupils completely dilated, eyes fixed and rolled upwards, with entire loss of vision, difficulty of deglution, paucity of urine, paralysis of extremities of right side, working of the mouth, frequent sighing, rapid thready pulse, and incessant coughing. Commenced giving Iodine according to following formula. ℞. Iod. Pot., ℈ij.; Aqua., ℥iv. M. Give a teaspoonful every two hours. After continuing this for 48 hours with material improvement, ʒjss. Iod. Pot. to ℥iv. Water was ordered in same doses; on the third day, the pupils were observed to contract partially; there was *very copious diuresis,* and the child began to notice her attendants, and swallowed readily. The dose was diminished, and after a farther continuance for eight days, with steady improvement, Iod. Ferri was substituted for Iod. Pot.

The child recovered, and is now healthy and grows finely.

